# Advanced Multimodal Imaging in Granulomatous Uveitis: From Differential Diagnosis to Treatment Monitoring and Surgical Integration

**DOI:** 10.3390/jcm15114222

**Published:** 2026-05-29

**Authors:** Matteo Capobianco, Francesco Bandello, Elisabetta Miserocchi, Federico Rissotto

**Affiliations:** 1 Department of Ophthalmology, IRCCS San Raffaele Scientific Institute, Vita-Salute San Raffaele University, Via Olgettina 60, 20132 Milan, Italy; bandello.francesco@hsr.it (F.B.); miserocchi.elisabetta@hsr.it (E.M.); 2 Eye Clinic, University of Catania, San Marco Hospital, 95121 Catania, Italy

**Keywords:** granulomatous uveitis, multimodal imaging, optical coherence tomography, indocyanine green angiography, ultrawidefield imaging, Vogt–Koyanagi–Harada disease, sarcoid uveitis, sympathetic ophthalmia, tuberculosis-associated uveitis, syphilitic uveitis

## Abstract

**Background/Objectives**: Granulomatous uveitis comprises a clinically heterogeneous group of inflammatory disorders, including ocular sarcoidosis, Vogt–Koyanagi–Harada disease, sympathetic ophthalmia, tuberculosis-associated uveitis, and syphilitic uveitis. Because these entities may share overlapping posterior segment findings, clinical examination alone is often insufficient for differential diagnosis, particularly when choroidal, retinal, or retinal vascular involvement predominates. **Methods**: This review provides a clinically oriented overview of multimodal imaging in granulomatous uveitis, including optical coherence tomography (OCT), enhanced-depth imaging OCT, swept-source OCT, OCT angiography, fundus autofluorescence, fluorescein angiography, indocyanine green angiography, and ultrawidefield imaging. **Results**: Emphasis is placed on imaging patterns that help localize the predominant anatomic compartment of inflammation, distinguish major etiologies, identify diagnostic pitfalls, and assess disease activity over time. By integrating current evidence with representative multimodal imaging findings, we propose an anatomic and decision-oriented framework for interpreting granulomatous posterior segment inflammation. **Conclusions**: Particular attention is given to the distinction between active inflammation and irreversible structural damage, as this distinction may influence treatment escalation or tapering, timing of elective surgery, local corticosteroid therapy, and the need for diagnostic sampling in infectious or masquerade-like presentations.

## 1. Introduction

Granulomatous uveitis includes a heterogeneous group of inflammatory disorders, such as ocular sarcoidosis, Vogt–Koyanagi–Harada (VKH) disease, sympathetic ophthalmia, tuberculosis-associated uveitis, and syphilitic uveitis. Clinically, granulomatous uveitis is typically suggested by large ‘mutton-fat’ keratic precipitates, iris or trabecular nodules, and, when the posterior segment is involved, by findings such as choroidal granulomas, multifocal choroiditis, retinal vasculitis, optic disc inflammation, or granulomatous chorioretinal lesions. Although these conditions differ in etiology, systemic context, and clinical behavior, they often share overlapping posterior segment features, which can make differential diagnosis challenging when based on clinical examination alone. In this context, multimodal imaging has become a central tool in modern uveitis practice, supporting diagnosis, differential diagnosis, evaluation of inflammatory activity, and treatment monitoring [1,2,3,4].

Beyond documenting lesion morphology, multimodal imaging helps clinicians define the predominant site of inflammation—retina, choriocapillaris, or choroidal stroma—identify peripheral or otherwise subtle abnormalities, and monitor structural and vascular changes during treatment. This is particularly relevant in granulomatous uveitis, where similar fundus appearances may correspond to distinct retinochoroidal patterns and different levels of disease activity. Ultrawidefield fundus photography and angiography extend the assessment of peripheral retinal signs and disease distribution, while ICGA, EDI/SS-OCT, and OCTA provide a more detailed evaluation of the choroid, outer retina, and choriocapillaris. Together, these techniques support a standardized multimodal approach, rather than reliance on any single imaging modality alone [5,6,7,8].

Compared with previous reviews that have broadly addressed multimodal imaging in infectious and noninfectious posterior uveitis, OCT angiography in inflammatory eye disease, or the general role of ultrawidefield angiography, this review focuses specifically on granulomatous uveitis as a clinically heterogeneous but imaging-relevant group of disorders [1,2,3,4,5,6,7,8]. Its main contribution is not to describe individual imaging signs in isolation, but to organize them into an anatomic and decision-oriented framework. In particular, we emphasize how multimodal imaging can help distinguish stromal choroiditis-dominant phenotypes from retinal, retinal pigment epithelial, choriocapillaris, or retinal vasculitis-dominant phenotypes; how imaging findings should be interpreted as active inflammation versus structural damage; and how this distinction may influence treatment monitoring, surgical timing, local therapy, and the need for diagnostic sampling.

Although granulomatous uveitis is often discussed mainly from a diagnostic perspective, advanced imaging also has direct therapeutic and surgical implications. OCT, FA, ICGA, FAF, OCTA, and ultrawidefield imaging may influence when to intensify systemic or local anti-inflammatory treatment, when to postpone elective surgery because inflammatory activity persists, when to proceed with cataract or vitreoretinal surgery after disease quiescence, and when diagnostic vitrectomy or tissue sampling should be considered in atypical, infectious, or masquerade-like presentations. For this reason, this review was designed not only as a diagnostic overview, but as a clinically oriented framework linking imaging phenotypes with treatment monitoring and procedural decision-making.

Importantly, granulomatous uveitis should not be regarded as a single homogeneous clinicopathologic entity. In this review, the term is used as a clinically oriented umbrella that includes disorders with different mechanisms and predominant anatomic targets. VKH disease and sympathetic ophthalmia are best interpreted as primary stromal choroiditis entities, whereas syphilitic uveitis more often involves the retina, retinal pigment epithelium, and retinal vasculature. Tuberculosis-associated uveitis may present as choroiditis, retinal vasculitis, or mixed retinochoroidal inflammation. Accordingly, this review adopts an anatomic and pathophysiology-based imaging framework rather than a purely disease-by-disease description.

Granulomatous uveitis can occur in several ocular and systemic contexts. Here, we focus on five entities selected because they are among the most clinically relevant, imaging-dependent, and diagnostically challenging causes of granulomatous posterior segment inflammation: ocular sarcoidosis, Vogt–Koyanagi–Harada disease, sympathetic ophthalmia, tuberculosis-associated uveitis, and syphilitic uveitis. Idiopathic granulomatous anterior or intermediate uveitis, lens-induced uveitis, fungal or parasitic granulomatous chorioretinitis, systemic autoimmune-associated granulomatous inflammation, and granulomatous masquerade syndromes are not reviewed as separate disease categories. Instead, they are discussed when relevant as mimickers, differential diagnoses, or triggers for diagnostic sampling. Therefore, the focus of this review is not granulomatous uveitis in its entirety, but the role of posterior segment multimodal imaging in clinically complex granulomatous or granulomatous-like uveitic entities.

## 2. Materials and Methods

A structured literature search was performed in PubMed/MEDLINE in April 2026, covering articles published from database inception to 30 April 2026. The search terms were used alone and in combination with Boolean operators and included: “granulomatous uveitis,” “ocular sarcoidosis,” “Vogt–Koyanagi–Harada disease,” “sympathetic ophthalmia,” “tuberculosis-associated uveitis,” “tubercular uveitis,” “syphilitic uveitis,” “multimodal imaging,” “optical coherence tomography,” “enhanced depth imaging OCT,” “swept-source OCT,” “fundus autofluorescence,” “fluorescein angiography,” “indocyanine green angiography,” “OCT angiography,” “ultrawidefield imaging,” “cataract surgery,” “vitrectomy,” “biopsy,” “uveitic macular edema,” and “steroid implant.”

The search aimed to identify peer-reviewed English-language articles addressing imaging findings, differential diagnosis, treatment monitoring, procedural planning, and surgical complications in granulomatous uveitis and related posterior segment inflammatory disorders. Priority was given to systematic reviews, meta-analyses, consensus statements, classification criteria, large cohort studies, randomized clinical trials, and clinically relevant imaging reviews. Case reports and small case series were included when they provided disease-specific multimodal imaging information not sufficiently addressed by larger studies, particularly for rare entities or infectious and masquerade presentations.

Articles were excluded when they did not address ocular imaging, were not clinically relevant to granulomatous uveitis or its main mimickers, focused only on anterior segment findings without posterior segment imaging implications, or lacked sufficient disease-specific information. The reference lists of selected articles were also screened manually to identify additional relevant publications.

Because this article was designed as a narrative review, and not as a systematic review or meta-analysis, the search was not prospectively registered. No formal PRISMA-based screening process or risk-of-bias assessment was performed. The final reference list included 66 articles, selected according to relevance, study design, journal quality, and contribution to imaging-based diagnosis, monitoring, or procedural decision-making in granulomatous uveitis.

All figures are original anonymized clinical images acquired during routine ophthalmic examinations at the Department of Ophthalmology, IRCCS San Raffaele Scientific Institute, Milan, Italy. No figures were reproduced or adapted from previously published material. Written informed consent was obtained at the time of clinical examination and image acquisition, including authorization for the anonymized scientific and educational use of clinical images.

## 3. Results

From a practical perspective, OCT plays a central role for structural assessment, as it detects serous retinal detachment, cystoid macular edema, epiretinal membrane, and outer retinal changes, while enhanced depth imaging or swept-source acquisition also allows evaluation of choroidal thickness and architecture. FA continues to play an important role in documenting retinal vascular leakage, optic disc leakage, macular ischemia or edema, and other angiographic signs of inflammatory activity, whereas ICGA is particularly useful when the main site of involvement is choroid or choriocapillaris. FAF adds valuable topographic information on retinal pigment epithelium fluorophores and outer retinal dysfunction, while OCTA provides a noninvasive view of retinal and choriocapillaris flow abnormalities as well as neovascular networks, although it does not replace dye-based angiography when leakage assessment is required [5,6,7,8]. However, OCTA should be interpreted as an adjunctive modality in uveitis because inflammatory media opacity, segmentation errors, projection artifacts, motion artifacts, signal attenuation, and inter-device variability may substantially affect image quality and quantitative measurements.

### 3.1. Ocular Sarcoidosis

In ocular sarcoidosis, imaging becomes especially valuable when the clinical picture is suggestive but not sufficiently specific on its own. The revised IWOS diagnostic criteria and the SUN classification criteria offer a structured framework, while posterior segment and peripheral imaging may reveal findings that support a sarcoid pattern, including nodular or segmental periphlebitis, peripheral vascular leakage or nonperfusion, multifocal choroiditis or choroidal nodules/granulomas, optic disc inflammation, and chronic structural complications such as cystoid macular edema or epiretinal membrane [9,10,11]. In this setting, FA, particularly UWF-FA, is useful for identifying retinal vasculitis and peripheral leakage, whereas ICGA and OCT, especially EDI-OCT, are helpful in characterizing deeper choroidal granulomatous involvement and associated retinal or outer retinal changes [12,13].

**Figure 1:**
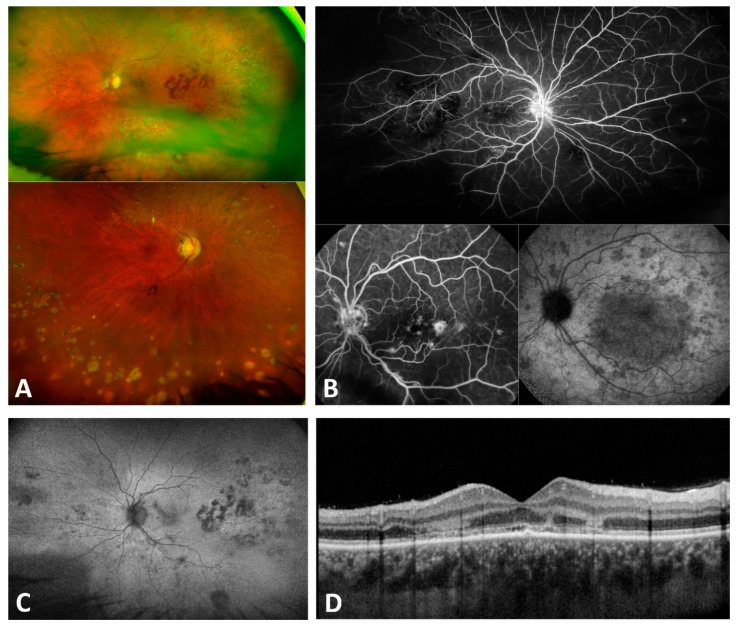
Multimodal imaging in ocular sarcoidosis. Composite figure assembled from original images of representative cases of active ocular sarcoidosis evaluated at IRCCS San Raffaele Scientific Institute, Milan. (**A**) Ultrawidefield color fundus photographs showing posterior and peripheral inflammatory lesions, including multifocal chorioretinal involvement and granulomatous-appearing lesions. (**B**) Fluorescein angiography demonstrating the retinal vasculitic component, with vascular leakage and areas of nonperfusion; the additional angiographic views highlight lesion distribution and the extent of vascular involvement. (**C**) Fundus autofluorescence showing topographic retinal pigment epithelium and outer retinal abnormalities corresponding to inflammatory lesions. (**D**) Optical coherence tomography demonstrating structural retinal and choroidal changes, including lesion-related elevation and outer retinal/RPE disruption. Overall, the figure illustrates the distinction between retinal vasculitic involvement, best appreciated on fluorescein angiography, and deeper granulomatous posterior segment disease, better characterized with multimodal structural imaging. Composite figure assembled from original anonymized clinical images obtained from representative cases evaluated at IRCCS San Raffaele Scientific Institute, Milan, Italy. The panels are intended to illustrate characteristic multimodal imaging findings and do not necessarily derive from the same eye or the same patient.

A major strength of multimodal imaging in sarcoid uveitis is its ability to clarify whether posterior inflammation is mainly retinal or whether substantial choroidal involvement is also present. On OCT, choroidal granulomas typically appear as hyporeflective elevated lesions, often associated with focal choroidal thickening, whereas on ICGA they are usually seen as hypocyanescent dark dots or lesions in the early and intermediate phases. By contrast, retinal vasculitis, peripheral nonperfusion, and peripheral neovascularization are often documented more completely on ultrawidefield fluorescein angiography than on routine clinical examination or conventional fluorescein angiography. Thus, in sarcoid uveitis, imaging interpretation should distinguish retinal periphlebitis-dominant disease, best assessed with FA or UWF-FA to define the retinal vasculitic component, from choroidal granulomatous involvement, for which ICGA and EDI/SS-OCT are more informative [12,13]. Representative multimodal imaging findings in ocular sarcoidosis are shown in Figure 1.

### 3.2. Vogt–Koyanagi–Harada Disease

VKH represents the prototype of bilateral primary stromal choroiditis within the spectrum of granulomatous uveitis, and its imaging phenotype shows particularly well why a multimodal approach is needed. Recent simplified diagnostic approaches and the SUN classification criteria emphasize a compatible bilateral presentation, characteristic posterior segment findings, and careful exclusion of relevant mimickers [14,15]. In the acute exudative phase, VKH is typically characterized by diffuse stromal choroidal inflammation, marked choroidal thickening, multifocal serous retinal detachments, and optic disc leakage. ICGA is central for detecting occult choroidal inflammation, especially hypocyanescent dark dots and diffuse stromal choroidal involvement, whereas EDI-OCT and SS-OCT provide complementary structural information on choroidal thickening, subretinal fluid, and anatomical recovery after treatment [14,16].

Longitudinal imaging is particularly important in VKH because choroidal inflammation may persist even when the clinical picture appears to improve. For this reason, ICGA and EDI/SS-OCT are especially useful during treatment tapering, helping to detect subclinical choroidal activity and to distinguish persistent inflammation from residual structural damage [14,16]. Early and adequate treatment during the initial inflammatory phase has also been proposed as a therapeutic window of opportunity, potentially reducing the risk of chronic recurrent disease and sunset-glow fundus [17]. Widefield SS-OCTA and quantitative OCTA studies suggest that active VKH may be associated with increased choroidal thickness and vascularity, together with reduced choriocapillaris flow; importantly, even eyes judged clinically inactive may show persistent choriocapillaris flow deficits or retinal microvascular abnormalities [18,19]. OCTA, however, should remain an adjunctive modality, since it does not assess leakage and cannot replace ICGA or dye-based angiography for evaluating inflammatory activity. In VKH, multimodal imaging is therefore most useful when ICGA and EDI/SS-OCT are used to assess stromal choroidal activity, while OCTA adds complementary information on retinal and choriocapillaris vascular changes. 

**Figure 2:**
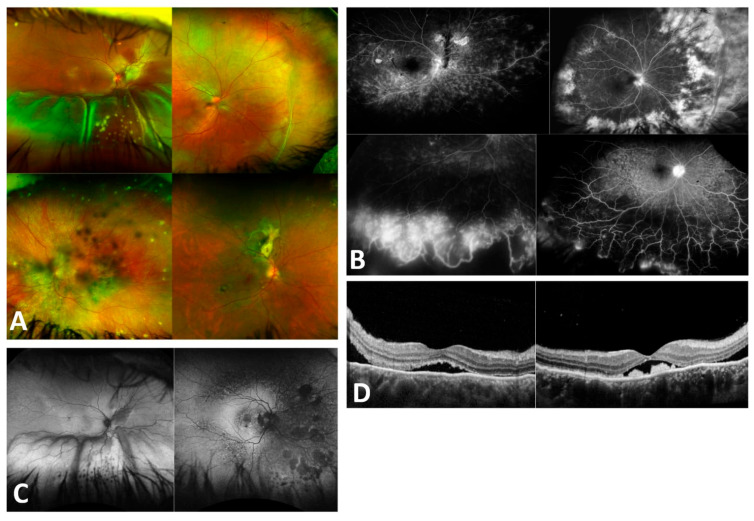
Multimodal imaging in Vogt–Koyanagi–Harada disease. Composite figure assembled from original images of representative cases of active VKH evaluated at IRCCS San Raffaele Scientific Institute, Milan. (**A**) Ultrawidefield color fundus photographs showing bilateral posterior segment involvement with multifocal exudative changes. (**B**) Angiographic views demonstrating active inflammatory leakage, including multifocal pinpoint leakage, pooling related to serous retinal detachment, and optic disc hyperfluorescence; these findings support active posterior segment inflammation. (**C**) Fundus autofluorescence showing areas of altered autofluorescence related to outer retinal and retinal pigment epithelium dysfunction. (**D**) Optical coherence tomography demonstrating serous retinal detachment and structural changes associated with diffuse choroidal inflammation. The figure highlights the typical multimodal pattern of active VKH, in which angiographic signs of inflammation and OCT evidence of exudative retinal detachment complement the assessment of stromal choroiditis. Composite figure assembled from original anonymized clinical images obtained from representative cases evaluated at IRCCS San Raffaele Scientific Institute, Milan, Italy. The panels are intended to illustrate characteristic multimodal imaging findings and do not necessarily derive from the same eye or the same patient.

Representative multimodal imaging findings in Vogt–Koyanagi–Harada disease are shown in Figure 2.

### 3.3. Sympathetic Ophthalmia

Sympathetic ophthalmia shares many imaging features with VKH, as both can present with bilateral uveitis and marked choroidal involvement, including serous retinal detachments, optic disc swelling, and, in some cases, late depigmentary fundus changes. The main distinguishing element, however, remains a history of unilateral penetrating trauma or intraocular surgery, which is central to the SUN classification criteria. In this setting, imaging helps demonstrate bilateral inflammation and confirm that the disease extends beyond isolated anterior uveitis, most often as anterior chamber and vitreous inflammation or panuveitis with choroidal involvement [20,21,22]. OCT and EDI-OCT may reveal serous retinal detachment, hyperreflective septa or bacillary layer detachment, choroidal thickening, folds, loss of normal choroidal architecture, and Dalen–Fuchs nodules, while FA and ICGA are useful for documenting active posterior inflammation and choroidal lesions [21,22].

Imaging findings in sympathetic ophthalmia substantially overlap with VKH; therefore, imaging supports activity assessment and follow-up, but the history of ocular trauma or intraocular surgery remains decisive for diagnosis. In the acute phase, imaging may show multifocal choroiditis, optic disc swelling, serous retinal detachments, and, less commonly, MEWDS-like changes or retinal vasculitis with chorioretinitis. In the chronic phase, more informative findings include widespread mid- and far-peripheral chorioretinal atrophy, peripapillary subretinal fibrosis, and nummular perivascular atrophy [20,21,22]. No single imaging sign is pathognomonic, but multimodal correlation is valuable both for supporting the diagnosis in the appropriate clinical context and for documenting persistent inflammatory activity or chronic structural damage over time [20,21,22,23]. Compared with VKH, sympathetic ophthalmia shows substantial overlap on multimodal imaging, including bilateral stromal choroiditis, serous retinal detachment, choroidal thickening, optic disc leakage, and ICGA choroidal lesions. Imaging alone rarely distinguishes the two entities with certainty. The clinical history of penetrating trauma or intraocular surgery remains decisive, while chronic findings such as peripheral chorioretinal atrophy, peripapillary subretinal fibrosis, and nummular perivascular atrophy may support the diagnosis in the appropriate context.

**Figure 3:**
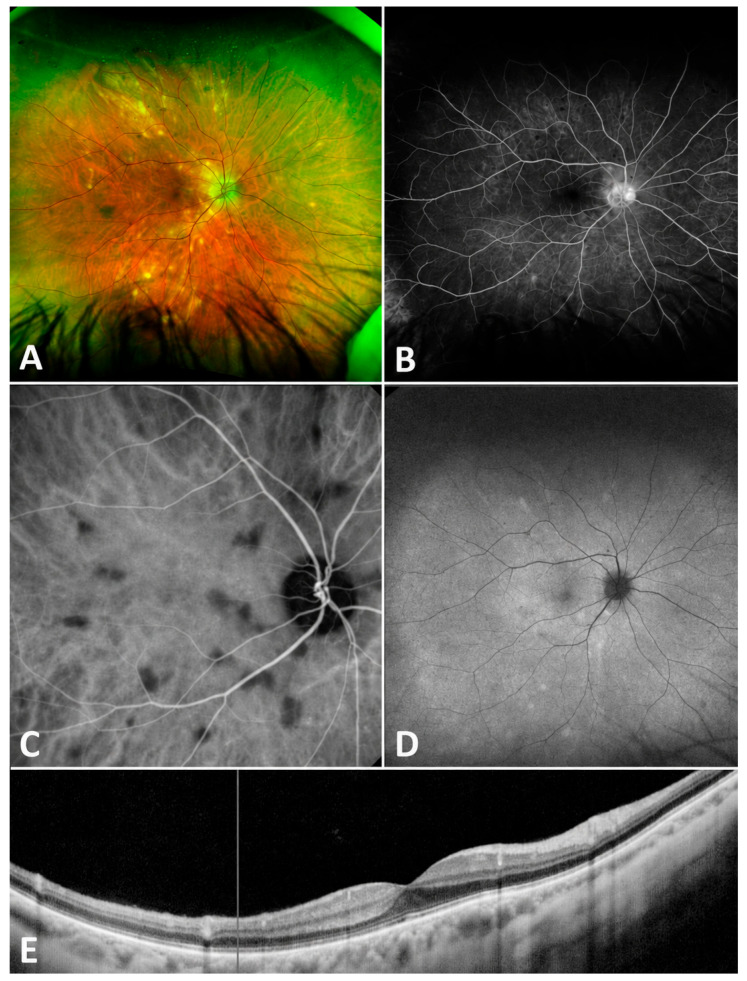
Multimodal imaging in sympathetic ophthalmia. Composite figure assembled from original images of representative cases of active sympathetic ophthalmia evaluated at IRCCS San Raffaele Scientific Institute, Milan, Italy. (**A**) Ultrawidefield color fundus photograph showing posterior segment inflammatory changes compatible with sympathetic ophthalmia. (**B**) Fluorescein angiography demonstrating active posterior segment inflammation, including optic disc leakage and lesion-related hyperfluorescence. (**C**) Indocyanine green angiography showing multiple hypocyanescent choroidal lesions, consistent with stromal choroidal inflammatory involvement. (**D**) Fundus autofluorescence showing retinal pigment epithelium disturbance related to active or chronic inflammatory damage. (**E**) Optical coherence tomography demonstrating serous retinal detachment, choroidal thickening, and associated structural inflammatory changes. This figure illustrates the substantial multimodal imaging overlap between sympathetic ophthalmia and Vogt–Koyanagi–Harada disease; therefore, imaging supports activity assessment and follow-up, but the history of penetrating ocular trauma or intraocular surgery remains essential for diagnosis. Composite figure assembled from original anonymized clinical images obtained from the same eye of the same patient evaluated at IRCCS San Raffaele Scientific Institute, Milan, Italy. The panels illustrate complementary multimodal imaging findings from the same clinical case.

Representative multimodal imaging findings in sympathetic ophthalmia are shown in Figure 3.

### 3.4. Tuberculosis-Associated Uveitis

Tuberculosis-associated uveitis remains diagnostically challenging because direct microbiologic confirmation from ocular tissues or fluids is only rarely obtained, so the diagnosis is still presumptive in many cases. Current consensus recommendations from the COTS group and the BTS clinical statement support a composite diagnostic approach based on a compatible ocular phenotype, corroborative immunologic and radiologic evidence of tuberculosis, exclusion of alternative causes, and multidisciplinary evaluation, rather than on direct ocular proof alone [24,25,26]. In this context, imaging does not establish the etiology by itself, but it plays a central role in phenotypic characterization, particularly in tubercular serpiginous-like choroiditis, tuberculoma, unifocal or multifocal choroiditis, retinal vasculitis, and posterior segment involvement more broadly. Imaging interpretation should be phenotype-specific: serpiginous-like choroiditis is best assessed with FAF, OCT, and ICGA to define lesion borders and activity; tuberculoma requires structural OCT and choroidal imaging; retinal vasculitis-dominant disease relies mainly on FA or UWF-FA; and panuveitis requires integration of posterior segment imaging with systemic and microbiological assessment [24,25,26,27,28].

In tuberculosis-associated uveitis, multimodal imaging is especially useful for defining lesion morphology, extent, activity, healing, and complications over time. Fundus photography and fundus autofluorescence (FAF) help document lesion borders and their evolution; optical coherence tomography (OCT) demonstrates outer retinal, retinal pigment epithelium (RPE), and choroidal involvement; indocyanine green angiography (ICGA) may reveal choroidal or choriocapillaris abnormalities that are more extensive than clinically apparent; and ultrawidefield (UWF) imaging can assess peripheral vasculitis, nonperfusion, neovascularization, or paradoxical worsening that may influence management [26,27]. Longitudinal data from the Collaborative Ocular Tuberculosis Study (COTS) also highlight the importance of serial follow-up, because treatment outcomes in ocular tuberculosis are ultimately judged by sustained control of inflammation and long-term inactivity after antitubercular therapy, rather than by baseline appearance alone [24,25,29].

**Figure 4:**
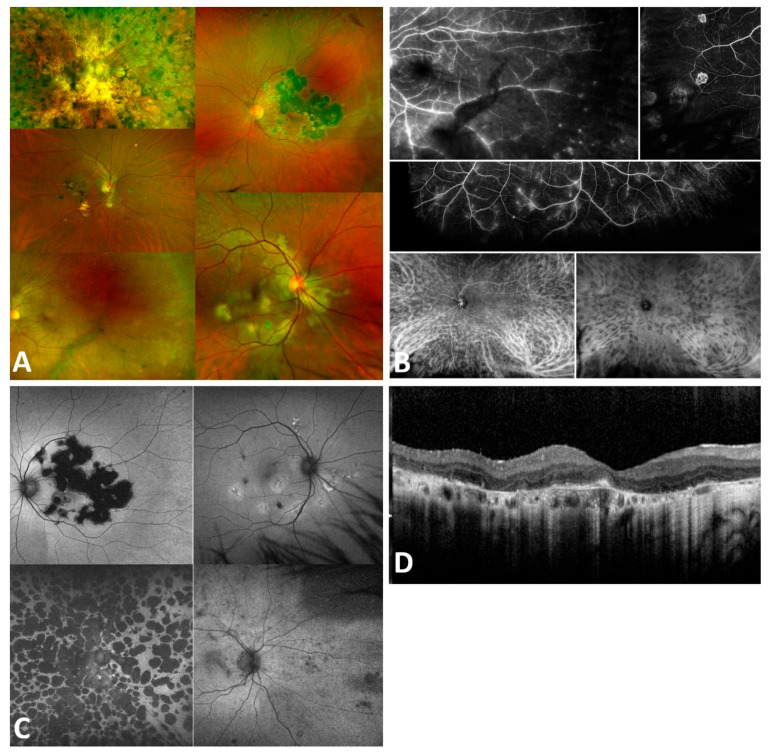
Multimodal imaging in tuberculosis-associated uveitis. Composite figure assembled from original images of representative cases of tuberculosis-associated uveitis evaluated at IRCCS San Raffaele Scientific Institute, Milan, illustrating different posterior segment phenotypes. (**A**) Ultrawidefield color fundus photographs showing representative posterior and peripheral lesions, including multifocal choroiditis/tuberculoma-like lesions and serpiginous-like choroidal involvement. (**B**) Angiographic views demonstrating retinal vasculitis, vascular leakage, nonperfusion, and inflammatory lesion activity; these images highlight the vascular phenotype and the extent of peripheral disease. (**C**) Fundus autofluorescence showing lesion borders and mixed autofluorescence patterns related to retinal pigment epithelium and outer retinal involvement, useful for assessing lesion activity and healing. (**D**) Optical coherence tomography demonstrating structural abnormalities involving the outer retina, retinal pigment epithelium, and choroid-overactive inflammatory lesions. Overall, the figure illustrates the phenotype-specific interpretation required in ocular tuberculosis, in which vasculitic, serpiginous-like, and granulomatous choroidal patterns may require different multimodal emphasis. Composite figure assembled from original anonymized clinical images obtained from representative cases evaluated at IRCCS San Raffaele Scientific Institute, Milan, Italy. The panels are intended to illustrate characteristic multimodal imaging findings and do not necessarily derive from the same eye or the same patient.

Representative multimodal imaging findings in tuberculosis-associated uveitis are shown in Figure 4.

### 3.5. Syphilitic Uveitis

Syphilitic uveitis remains one of the classic great masqueraders and should be considered in the differential diagnosis of virtually any uveitic presentation, particularly posterior uveitis and panuveitis. The SUN classification criteria emphasize that syphilitic uveitis can involve any anatomic class of uveitis and require a compatible ocular presentation together with evidence of Treponema pallidum infection, centered on a positive treponemal test within the recommended serologic algorithm. In posterior or panuveitic disease, imaging suggests that syphilis most often affects the retina, retinal pigment epithelium, or retinal vasculature, with recognizable patterns that include placoid inflammation at the level of the retinal pigment epithelium, multifocal retinal or retinal pigment epithelial inflammation, necrotizing retinitis, and retinal vasculitis [30]. Among these, acute syphilitic posterior placoid chorioretinitis (ASPPC) is the most characteristic posterior phenotype and the one in which multimodal imaging is particularly informative [31,32,33,34,35]. However, syphilitic uveitis is broader than ASPPC and may also present with retinal vasculitis, necrotizing retinitis, punctate inner retinitis, multifocal retinitis, or placoid and white-dot-like inflammatory patterns. These appearances may mimic APMPPE, MEWDS/AZOOR-spectrum disorders, viral retinitis, inflammatory choriocapillaritis, or other posterior uveitides. For this reason, imaging findings should be considered suggestive but never diagnostic in isolation, and serologic confirmation should not be delayed when syphilis is clinically suspected. In ASPPC, multimodal imaging typically reveals a yellow placoid lesion at the posterior pole, hyperautofluorescence on fundus autofluorescence, disruption of the outer retina and ellipsoid zone with irregular or granular RPE changes on OCT, progressive late hyperfluorescence on fluorescein angiography, and hypofluorescence on late-phase ICGA. These abnormalities usually improve after prompt antibiotic treatment, and OCT may document substantial restoration of outer retinal architecture, reinforcing the value of imaging for both recognition and follow-up [32,33,35]. Even in placoid syphilis, however, imaging findings are not sufficient in isolation, because syphilitic posterior uveitis can mimic several inflammatory or infectious phenotypes. For this reason, multimodal imaging should always be interpreted alongside serologic testing and within the broader clinical context [30,31,32,33,34,35].

**Figure 5:**
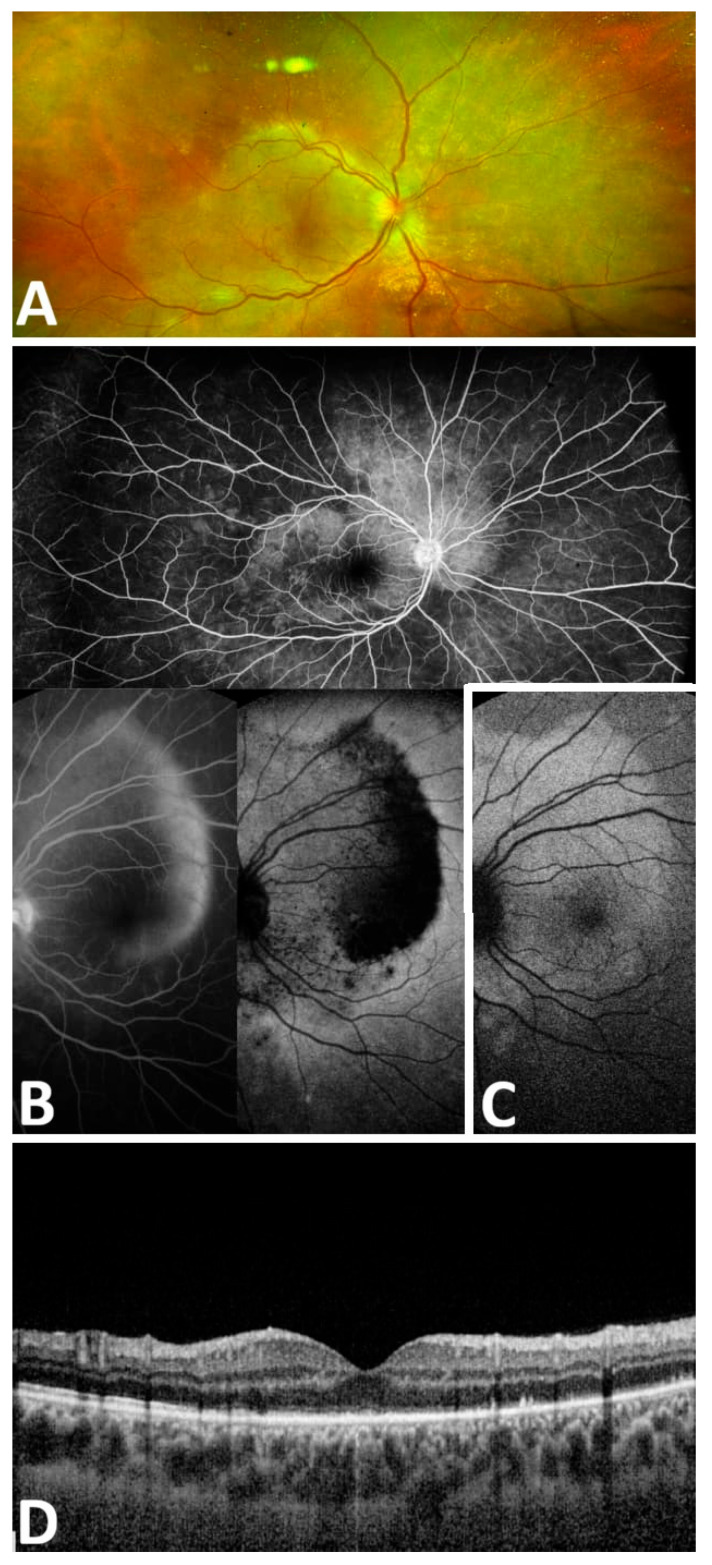
Multimodal imaging in acute syphilitic posterior placoid chorioretinitis (ASPPC). Original image set from a representative case evaluated at IRCCS San Raffaele Scientific Institute, Milan. (**A**) Ultrawidefield color fundus photograph showing a placoid yellowish lesion centered at the posterior pole. (**B**) Fluorescein angiography demonstrating lesion-related hyperfluorescence, with progressive late staining of the placoid area. (**C**) Fundus autofluorescence showing hyperautofluorescence corresponding to the placoid lesion. (**D**) Optical coherence tomography demonstrating disruption of the outer retina and ellipsoid zone, with irregular retinal pigment epithelium changes corresponding to the placoid lesion. Together, these findings illustrate the characteristic multimodal correlation of ASPPC, in which the posterior placoid fundus lesion corresponds to outer retinal/RPE disruption on OCT and a distinctive angiographic/autofluorescence pattern. Composite figure assembled from original anonymized clinical images obtained from the same eye of the same patient evaluated at IRCCS San Raffaele Scientific Institute, Milan, Italy. The panels illustrate complementary multimodal imaging findings from the same clinical case.

Representative multimodal imaging findings in acute syphilitic posterior placoid chorioretinitis are shown in Figure 5.

### 3.6. Differential-Diagnostic Pitfalls and Overlapping Imaging Patterns

Several multimodal imaging findings in granulomatous uveitis are clinically suggestive, but rarely disease-specific. Choroidal thickening, serous retinal detachment, hypocyanescent ICGA lesions, outer retinal disruption, vascular leakage, and FAF abnormalities may be shared by different inflammatory and infectious entities. For this reason, the diagnostic value of imaging does not rest on isolated signs, but on the combined interpretation of the predominant anatomic compartment, lesion distribution, angiographic behavior, laterality, clinical history, and laboratory context.

In stromal choroiditis-dominant phenotypes, VKH and sympathetic ophthalmia may show considerable overlap, including diffuse choroidal thickening, serous retinal detachment, optic disc leakage, and hypocyanescent lesions on ICGA. In this setting, ICGA and EDI/SS-OCT are useful to document occult choroidal activity and to monitor persistence or recurrence over time. However, imaging alone cannot reliably separate these two entities; a history of penetrating ocular trauma or intraocular surgery remains the key element supporting sympathetic ophthalmia [14,16,20,21,22,23]. By contrast, retinal vasculitis-dominant disease is better assessed with FA and UWF-FA, whereas choroidal granulomas, tuberculoma, and serpiginous-like choroiditis require integration of OCT, ICGA, FAF, and clinical or microbiological correlation [12,24,25,26,27,28,29].

Syphilitic uveitis is another important diagnostic pitfall. ASPPC may display a recognizable multimodal imaging pattern, but placoid abnormalities involving the outer retina or RPE can also be seen in non-syphilitic inflammatory chorioretinopathies and white-dot-like disorders. Imaging can therefore raise suspicion, but it cannot establish the diagnosis on its own, and serologic confirmation remains mandatory. When ocular syphilis is clinically suspected, imaging should help with recognition and follow-up, but it should never delay appropriate antimicrobial treatment [30,31,32,33,34,35].

For ease of clinical application, a condensed overview of the main imaging patterns, most informative modalities, key mimickers, and activity-related features across the major granulomatous uveitic entities is provided in Table 1. The expanded modality-by-modality version is provided as Supplementary Table S1.

## 4. Disease-Specific Imaging-Guided Treatment Monitoring and Procedural Decision-Making

In this review, surgical and procedural implications are considered only insofar as they are guided by disease-specific multimodal imaging findings in granulomatous uveitis. The aim of this section is therefore not to provide a general overview of uveitic surgery, but to clarify how imaging may influence treatment escalation, timing of elective surgery, local corticosteroid therapy, diagnostic sampling, or vitreoretinal intervention in the main entities discussed in this review. In ocular sarcoidosis, the key issue is often to distinguish retinal vasculitic activity from granulomatous choroidal involvement. In VKH disease and sympathetic ophthalmia, imaging is particularly useful for identifying persistent stromal choroiditis or chronic structural sequelae. In tuberculosis-associated and syphilitic uveitis, imaging may suggest inflammatory activity or complications, but corticosteroid escalation, steroid implants, or perioperative anti-inflammatory strategies should be considered cautiously when infection is active, suspected, or insufficiently excluded, and should be integrated with appropriate infectious evaluation and antimicrobial management.

### 4.1. Imaging-Guided Elective Surgery and Cataract Planning

Before elective ocular surgery, multimodal imaging should complement, rather than replace, standardized clinical assessment of inflammatory activity. The SUN Working Group emphasized the importance of consistent terminology, inflammatory grading, and separate documentation of structural complications in uveitis [36]. This distinction is especially relevant in granulomatous uveitis, because reduced visual potential may reflect either active inflammation or irreversible sequelae such as macular atrophy, chronic cystoid macular edema, epiretinal membrane, optic atrophy, retinal ischemia, RPE damage, or chorioretinal scarring [37,38].

From a disease-specific perspective, preoperative imaging has different implications across the entities reviewed. In ocular sarcoidosis, OCT may identify CME, ERM, or macular structural damage, whereas FA or UWF-FA can reveal persistent retinal vascular leakage, peripheral nonperfusion, or neovascularization that may require treatment before elective surgery. ICGA and EDI/SS-OCT may be useful when granulomatous choroidal involvement is suspected. In VKH disease and sympathetic ophthalmia, ICGA and EDI/SS-OCT are particularly relevant because clinically quiet eyes may still show persistent stromal choroidal activity, while chronic RPE, outer retinal, or chorioretinal damage may limit postoperative visual recovery without necessarily indicating active disease [14,16,20,21,22,23]. In tuberculosis-associated and syphilitic uveitis, imaging abnormalities should be interpreted together with systemic, microbiological, and serological evaluation before perioperative corticosteroid escalation is considered [24,25,26,27,28,29,30].

Whenever clinically feasible, cataract surgery in uveitic eyes should be performed after a period of inflammatory quiescence, commonly at least three months, because recent inflammatory activity is associated with a higher risk of postoperative inflammation and CME [37,38,39]. OCT remains the most practical tool for detecting pre-existing or postoperative CME, while FA or UWF-FA may be useful when persistent retinal vascular leakage, ischemia, or peripheral inflammatory activity is suspected [5,38,39,40]. If dense cataract or media opacity prevents adequate fundus visualization, B-scan ultrasonography may help exclude retinal detachment, significant vitritis, or mass-like posterior segment abnormalities before surgery [38]. Although large cohorts and trial-based data show that cataract surgery can improve vision in uveitic eyes, outcomes remain less predictable than in non-uveitic eyes because of uveitis-related comorbidities and postoperative complications [41,42,43,44]. In granulomatous uveitis, the practical role of imaging is therefore to identify active, treatable inflammation, estimate visual potential, and support individualized perioperative planning.

### 4.2. Diagnostic Vitrectomy, Biopsy, and Infectious or Masquerade Work-Up

Diagnostic vitrectomy or tissue sampling should not be considered a routine step in granulomatous uveitis. Its role is more specific: it becomes relevant when the clinical course, imaging pattern, or treatment response raises concern for infection, intraocular malignancy, or another masquerade syndrome. Multimodal imaging can help define whether the dominant abnormality involves the vitreous, retina, RPE, choroid, or retinal vasculature, and can therefore guide the choice between aqueous sampling, vitreous sampling, chorioretinal biopsy, or systemic tissue evaluation [45,46,47,48,49,50,51,52].

The threshold for diagnostic sampling should be lower in atypical, severe, progressive, or treatment-refractory presentations. Imaging findings that may prompt this approach include dense or persistent vitritis of uncertain origin, necrotizing retinitis, progressive chorioretinal or RPE lesions despite treatment, atypical placoid or infiltrative lesions, unexplained recurrent vasculitis, subretinal or sub-RPE deposits, choroidal masses or infiltrates, and discordance between ocular imaging and systemic work-up [45,46,47,48,49,50]. In ocular sarcoidosis and tuberculosis-associated uveitis, choroidal granulomatous lesions or mass-like infiltrates may overlap clinically and radiologically with infectious, inflammatory, neoplastic, or other masquerade entities. In syphilitic uveitis, placoid or white-dot-like inflammatory patterns may mimic noninfectious choriocapillaritis, viral retinitis, or lymphoma; therefore, imaging should support recognition and follow-up but should not delay serologic confirmation or antimicrobial treatment [30,31,32,33,34,35].

When primary vitreoretinal lymphoma or another malignant masquerade is suspected, imaging may strengthen suspicion but cannot establish the diagnosis. OCT may show sub-RPE or pre-Bruch’s deposits and outer retinal or RPE abnormalities, FAF may reveal hyper- or hypoautofluorescent changes, and FA may show hypofluorescent lesions or variable leakage [49,50]. In such cases, diagnostic yield depends on careful coordination with the laboratory, adequate sample handling, and appropriate use of cytology, microbiology, PCR, flow cytometry, cytokine analysis, and molecular testing [45,46,47,48,49,50,51,52]. Thus, in granulomatous or granulomatous-like uveitis, multimodal imaging should be used to identify cases in which escalation of corticosteroids or immunosuppression would be unsafe without first excluding infection or malignancy.

### 4.3. Imaging-Guided Local Corticosteroid Therapy

Local corticosteroid therapy, including intravitreal dexamethasone implants and fluocinolone acetonide implants or inserts, may be useful in selected patients with noninfectious intermediate, posterior, or panuveitis, particularly when uveitic macular edema is present [53,54,55,56,57,58]. In granulomatous uveitis, however, imaging response must always be interpreted in relation to the underlying etiology. Steroid implants may be appropriate in noninfectious disease or after infection has been adequately treated and controlled, but they should not replace antimicrobial therapy in active, suspected, or insufficiently excluded tuberculosis-associated or syphilitic uveitis [24,25,26,27,28,29,30].

OCT is the main imaging modality for monitoring macular edema after local steroid therapy, including reduction in central macular thickness, resolution of intraretinal cysts or subretinal fluid, and partial restoration of outer retinal architecture when damage is reversible [53,54]. However, OCT improvement alone does not necessarily indicate complete inflammatory control. In ocular sarcoidosis, FA or UWF-FA may still show retinal vascular leakage, peripheral nonperfusion, or neovascularization despite macular improvement. In VKH disease and sympathetic ophthalmia, ICGA and EDI/SS-OCT may remain necessary to determine whether stromal choroidal inflammation has resolved [14,16,20,21,22,23]. Therefore, imaging-guided local therapy should be based on integrated multimodal assessment rather than on macular OCT endpoints alone.

### 4.4. Vitreoretinal Surgery and Postoperative Imaging Follow-Up

Vitreoretinal surgery in granulomatous uveitis may be considered for selected diagnostic or therapeutic indications, including non-clearing vitreous opacities, diagnostic uncertainty, suspected infectious or masquerade disease, epiretinal membrane, vitreomacular traction, macular hole, retinal detachment, vitreous hemorrhage, or complications related to retinal ischemia and neovascularization [45,46,47,59]. The relevance of multimodal imaging lies in determining whether surgery is being performed in an eye with controlled inflammation, active disease requiring treatment optimization, or irreversible structural damage that may limit visual recovery.

Preoperative OCT helps distinguish inflammatory macular edema from tractional maculopathy, ERM-related distortion, macular hole, or outer retinal loss. FA and UWF-FA can identify retinal vasculitis, ischemia, peripheral nonperfusion, or neovascularization, which may require perioperative anti-inflammatory treatment, laser photocoagulation, anti-VEGF therapy, or closer postoperative surveillance [5,45,59]. In sarcoidosis, this is particularly relevant when retinal vasculitis or peripheral ischemia predominates. In VKH disease and sympathetic ophthalmia, surgery should be planned with attention to persistent choroidal inflammatory activity and chronic chorioretinal sequelae. In tuberculosis-associated and syphilitic uveitis, infectious activity should be appropriately evaluated and treated before corticosteroid-based perioperative strategies are intensified [24,25,26,27,28,29,30].

After surgery, multimodal imaging remains useful because poor visual recovery may reflect recurrent CME, persistent vascular leakage, ERM recurrence, macular ischemia, outer retinal loss, or chorioretinal atrophy rather than surgical failure alone. Sympathetic ophthalmia should also remain in the differential diagnosis of bilateral postoperative inflammation after penetrating ocular trauma or intraocular surgery, including vitreoretinal procedures; in this setting, imaging documents bilateral choroidal inflammation, serous retinal detachment, optic disc leakage, or chronic chorioretinal sequelae, but clinical history remains essential for diagnosis [20,21,22,23,60].

Overall, the procedural value of multimodal imaging in granulomatous uveitis lies in its ability to identify which eyes have active, treatable inflammation, which have irreversible structural damage, and which require infectious or masquerade work-up before corticosteroid escalation or surgery.

## 5. Discussion

Across the granulomatous uveitic entities discussed in this review, the real clinical value of multimodal imaging lies less in the search for a single pathognomonic sign than in the recognition of reproducible imaging patterns. Bilateral stromal choroiditis, diffuse choroidal thickening, serous retinal detachment, and optic disc involvement point toward VKH or sympathetic ophthalmia; however, in the latter, the diagnosis remains closely tied to a history of ocular trauma or intraocular surgery [16,20,21,22,23]. Posterior placoid lesions with predominant involvement of the outer retina and RPE are more suggestive of syphilitic uveitis, particularly ASPPC, although serologic confirmation is still mandatory [30,31,32,33,34,35]. Multifocal granulomatous choroidal lesions may occur in both sarcoidosis and tuberculosis-associated uveitis, while a serpiginous-like pattern should raise suspicion for ocular tuberculosis when the clinical context is compatible [12,24,25,26,27,28,29].

The most useful way to read these findings in practice is therefore to combine an anatomic approach with an assessment of inflammatory activity. FA and UWF-FA are particularly valuable for detecting retinal vasculitis, vascular leakage, ischemia, and peripheral nonperfusion [61,62,63]. By contrast, ICGA and EDI/SS-OCT become more informative when stromal choroiditis or granulomatous choroidal involvement is suspected [5,6,7,8,14,16]. OCT remains indispensable for identifying CME, subretinal fluid, ERM, vitreomacular traction, and outer retinal damage. However, an improvement in retinal structure on OCT should not be equated automatically with complete inflammatory control, especially if angiographic leakage or choroidal activity persists.

This separation between ongoing inflammation and irreversible structural damage is central both to treatment monitoring and to surgical decision-making. Persistent CME, active vascular leakage, progressive FAF abnormalities, or active choroidal lesions should lead to optimization of systemic, antimicrobial, or local anti-inflammatory therapy before elective surgery whenever possible. Conversely, stable chorioretinal scars, RPE atrophy, outer retinal loss, optic atrophy, or fibrotic sequelae may account for limited visual recovery without necessarily indicating persistent disease activity [36,37,38,39,40,41,42,43,44]. In atypical, severe, or treatment-refractory cases, multimodal imaging can also help identify those situations in which diagnostic vitrectomy, biopsy, or intraocular fluid analysis is needed to exclude infection or masquerade syndromes [45,46,47,48,49,50,51,52].

OCTA and quantitative imaging are promising adjuncts, but their current clinical role remains limited by segmentation errors, projection artifacts, media opacity, motion artifacts, signal attenuation from inflammatory lesions, inter-device variability, and the lack of validated disease-specific thresholds [3,7,64,65,66]. For this reason, OCTA-derived metrics should not yet be used as isolated decision-making tools in granulomatous uveitis. Their greatest value at present is complementary: they may help characterize retinal and choriocapillaris flow abnormalities, but they do not replace dye-based angiography when leakage, vasculitis, or inflammatory activity must be assessed.

This review has limitations. It is a narrative review rather than a systematic review or meta-analysis, and no formal risk-of-bias assessment was performed. In addition, the available evidence is heterogeneous across diseases: VKH, ocular tuberculosis, and uveitic cataract surgery are supported by stronger consensus, cohort, or trial-based data, whereas sympathetic ophthalmia, rare infectious phenotypes, and masquerade-like presentations often rely on smaller series and expert interpretation. Nevertheless, by integrating diagnosis, monitoring, and procedural decision-making, this review proposes a practical framework for applying multimodal imaging in clinically complex granulomatous uveitis.

**Figure 6:**
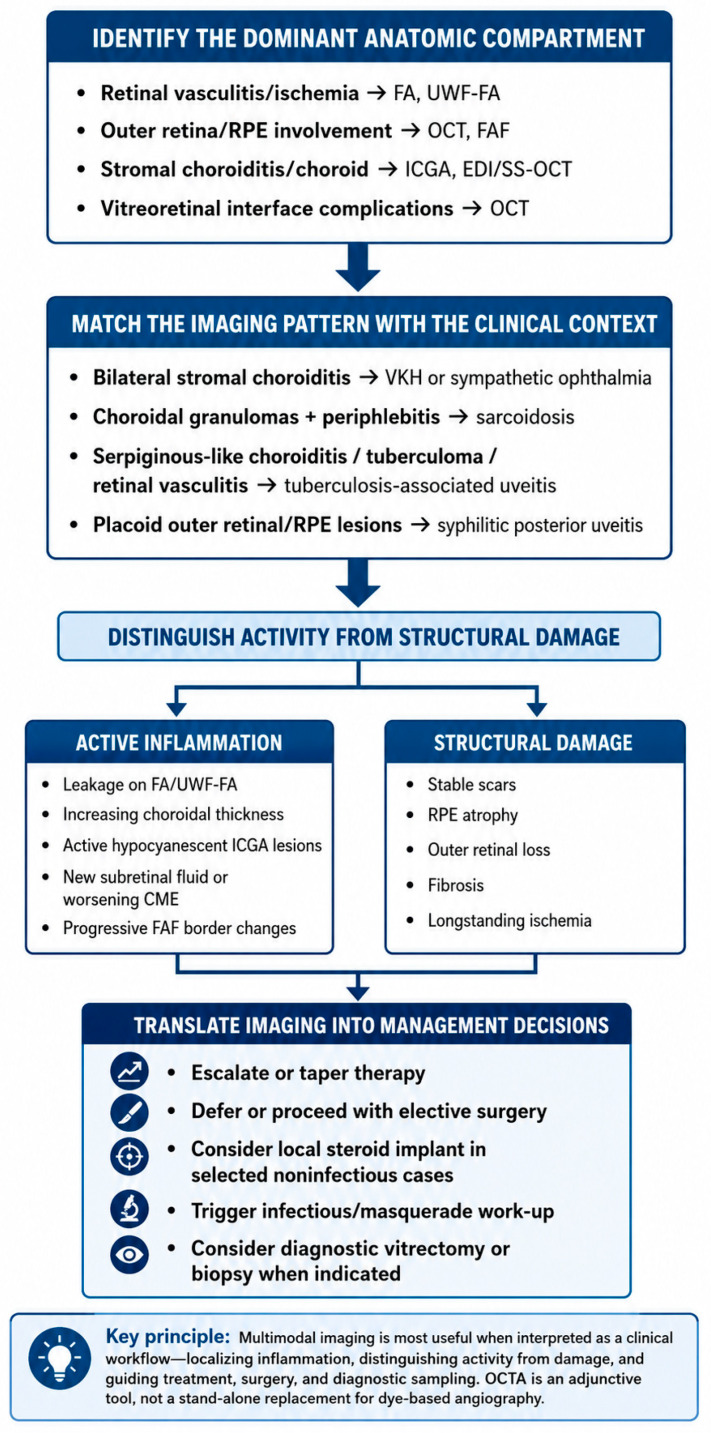
Proposed imaging-guided clinical workflow for granulomatous uveitis. Multimodal imaging is interpreted sequentially by identifying the dominant anatomic compartment, matching the imaging pattern with the clinical context, distinguishing active inflammation from structural damage, and translating these findings into treatment or procedural decisions.

To translate these concepts into a practical clinical workflow, we propose a stepwise imaging-guided approach for granulomatous uveitis, integrating anatomic localization, pattern recognition, activity assessment, and treatment or procedural decision-making (Figure 6).

## 6. Conclusions

Multimodal imaging in granulomatous uveitis should not be viewed only as a diagnostic adjunct. When interpreted through an anatomic and pattern-based framework, advanced imaging helps localize inflammation, distinguish activity from structural damage, identify disease-specific mimickers, guide treatment escalation or tapering, and support surgical or procedural decision-making. Its clinical value is particularly evident when imaging findings influence the timing of elective surgery, the need for diagnostic vitrectomy or biopsy, the monitoring of local corticosteroid implants, or the management of inflammatory complications such as macular edema, retinal vasculitis, ischemia, or tractional sequelae. OCTA and quantitative imaging remain promising adjuncts, but current clinical decisions should continue to rely on integrated multimodal interpretation rather than isolated quantitative parameters.

## Figures and Tables

**Table 1:** Condensed practical imaging clues for diagnosis and monitoring of major granulomatous uveitic entities.

Disease Entity	Dominant Imaging Pattern	Most Informative Modalities	Key Mimickers/Diagnostic Pitfalls	Active Inflammation vs. Structural Damage
**Ocular sarcoidosis**	Retinal periphlebitis or vasculitis with possible granulomatous choroidal involvement. Choroidal granulomas, multifocal choroiditis, optic disc leakage, CME, ERM, and peripheral vascular abnormalities may be present.	FA/UWF-FA to assess retinal vasculitis, leakage, nonperfusion, and peripheral neovascularization. ICGA and EDI/SS-OCT to characterize choroidal granulomas or occult choroidal involvement. OCT for CME, ERM, outer retinal changes, and structural complications.	Tuberculosis-associated choroiditis or tuberculoma, idiopathic multifocal choroiditis, intraocular lymphoma, choroidal metastasis, and other granulomatous choroidal lesions. Imaging supports the diagnosis only in the appropriate systemic and laboratory context.	Active disease: vascular leakage, optic disc leakage, CME, active or enlarging choroidal granulomas, ICGA hypocyanescent lesions. Inactive/damage: stable chorioretinal scars, RPE atrophy, chronic ERM, outer retinal loss, resolved leakage.
**Vogt–Koyanagi–Harada disease**	Bilateral primary stromal choroiditis with diffuse choroidal thickening, multifocal serous retinal detachments, optic disc leakage, and possible chronic RPE or outer retinal damage.	ICGA to detect stromal choroiditis and subclinical choroidal activity. EDI-OCT/SS-OCT to assess choroidal thickness, subretinal fluid, serous retinal detachment, and anatomical recovery. FA for pinpoint leakage, pooling, and disc leakage. OCTA may provide adjunctive information on choriocapillaris flow, but does not replace dye-based angiography.	Sympathetic ophthalmia, posterior scleritis, central serous chorioretinopathy, APMPPE-like disease, inflammatory choriocapillaritis, and other causes of bilateral exudative retinal detachment. Clinical history and systemic context remain essential.	Active disease: subretinal fluid, diffuse choroidal thickening, ICGA dark dots, optic disc leakage, progressive RPE or outer retinal changes. Inactive/damage: resolved fluid, reduced choroidal activity, persistent RPE disturbance, outer retinal loss, chorioretinal atrophy, sunset-glow fundus.
**Sympathetic ophthalmia**	Bilateral stromal choroiditis or panuveitis occurring after penetrating ocular trauma or intraocular surgery. Imaging may overlap substantially with VKH, including serous retinal detachment, choroidal thickening, optic disc leakage, and ICGA choroidal lesions.	OCT/EDI-OCT to assess serous retinal detachment, choroidal thickening, choroidal folds, and outer retinal changes. FA and ICGA to document active posterior inflammation and choroidal lesions. FAF may help identify chronic RPE disturbance, nummular atrophy, or chorioretinal sequelae.	VKH is the main diagnostic mimic. Sarcoidosis, tuberculosis-associated choroiditis, syphilitic panuveitis, posterior scleritis, and other bilateral granulomatous panuveitides should also be considered. Imaging alone rarely distinguishes sympathetic ophthalmia from VKH; the history of trauma or surgery is decisive.	Active disease: serous retinal detachment, choroidal thickening, optic disc leakage, ICGA choroidal lesions, active panuveitis. Inactive/damage: nummular chorioretinal atrophy, peripapillary subretinal fibrosis, peripheral chorioretinal atrophy, RPE or outer retinal loss.
**Tuberculosis-associated uveitis**	Phenotype-dependent pattern including serpiginous-like choroiditis, tuberculoma, multifocal choroiditis, retinal vasculitis, panuveitis, or mixed retinochoroidal inflammation. Imaging helps define phenotype but does not establish tubercular etiology alone.	FA/UWF-FA for retinal vasculitis, leakage, nonperfusion, and peripheral neovascularization. FAF and OCT for lesion borders, outer retinal/RPE involvement, and healing. ICGA for choroidal or choriocapillaris involvement, often more extensive than clinically visible disease.	Serpiginous choroiditis, ampiginous choroiditis, sarcoid choroidal granuloma, syphilitic chorioretinitis, fungal chorioretinitis, lymphoma, metastasis, and noninfectious retinal vasculitis. Diagnosis requires integration with systemic, immunologic, radiologic, and microbiological data.	Active disease: expanding lesion borders, active FAF changes, outer retinal/RPE disruption at lesion margins, vascular leakage, nonperfusion, neovascularization, vitritis, or paradoxical worsening. Inactive/damage: sharply demarcated scars, hypoautofluorescent atrophy, resolved leakage, stable chorioretinal scarring, RPE or outer retinal loss.
**Syphilitic uveitis**	Predominant involvement of the retina, RPE, outer retina, and retinal vasculature. ASPPC is the most characteristic posterior phenotype, with placoid posterior pole lesions, outer retinal/RPE disruption, and distinctive FAF/angiographic abnormalities.	OCT for ellipsoid zone disruption, outer retinal abnormalities, granular or irregular RPE changes, and recovery after treatment. FAF for hyperautofluorescent placoid lesions. FA for late hyperfluorescence, disc leakage, retinal vasculitis, or vascular leakage. ICGA may show late hypofluorescence of placoid lesions.	APMPPE, MEWDS/AZOOR-spectrum disorders, viral retinitis, inflammatory choriocapillaritis, tuberculosis-associated choroiditis, VKH-like disease, lymphoma, and other masquerade inflammatory phenotypes. Imaging is suggestive but never diagnostic; serologic confirmation is mandatory.	Active disease: placoid lesion, hyper-FAF, outer retinal/EZ disruption, FA leakage or late hyperfluorescence, ICGA hypofluorescence, retinal vasculitis, retinitis, or necrotizing retinitis. Inactive/damage: restoration of outer retinal layers when reversible; persistent RPE disturbance, outer retinal loss, chorioretinal atrophy, or residual FAF abnormalities.

Abbreviations: APMPPE, acute posterior multifocal placoid pigment epitheliopathy; ASPPC, acute syphilitic posterior placoid chorioretinitis; AZOOR, acute zonal occult outer retinopathy; CME, cystoid macular edema; EDI-OCT, enhanced-depth imaging optical coherence tomography; ERM, epiretinal membrane; EZ, ellipsoid zone; FA, fluorescein angiography; FAF, fundus autofluorescence; ICGA, indocyanine green angiography; MEWDS, multiple evanescent white dot syndrome; OCT, optical coherence tomography; OCTA, optical coherence tomography angiography; RPE, retinal pigment epithelium; SS-OCT, swept-source optical coherence tomography; UWF-FA, ultrawidefield fluorescein angiography; VKH, Vogt–Koyanagi–Harada disease.

## Data Availability

Data are contained within the article.

## Supplementary Materials

The following supporting information can be downloaded at: https://www.mdpi.com/article/10.3390/jcm15114222/s1, Table S1: Practical multimodal imaging clues in the differential diagnosis and follow-up of major granulomatous uveitic entities.

## Abbreviations

OCT	optical coherence tomography
EDI-OCT	enhanced-depth imaging OCT
SS-OCT	swept-source OCT
OCTA	OCT angiography
FAF	fundus autofluorescence
FA	fluorescein angiography
ICGA	indocyanine green angiography
UWF	ultrawidefield
VKH disease	Vogt–Koyanagi–Harada disease
ASPPC	acute syphilitic posterior placoid chorioretinitis
CME	cystoid macular edema
ERM	epiretinal membrane
RPE	retinal pigment epithelium
SUN	Standardization of Uveitis Nomenclature
IWOS	International Workshop on Ocular Sarcoidosis
COTS	Collaborative Ocular Tuberculosis Study
