# Supplementary material for: Advanced Multimodal Imaging in Granulomatous Uveitis: From Differential Diagnosis to Treatment Monitoring and Surgical Integration
Source: J Clin Med. 2026 May 29;15(11):4222. doi: 10.3390/jcm15114222 (PMC13258472; doi:10.3390/jcm15114222)
Supplement: Supplementary file 1 [file jcm-15-04222-s001.zip › jcm-4292183-supplementary.pdf]

Journal of
Clinical Medicine

MDPI

Table S1. Practical multimodal imaging clues in the differential diagnosis and follow-up of major granulomatous uveitic entities.

Disease entity	Primary anatomic site of inflammation	Key OCT findings	Key FA findings	Key ICGA findings	Key FAF findings	OCTA findings and limitations	Imaging clues favoring diagnosis	Important mimickers	Imaging features of active vs inactive disease
**Ocular sarcoidosis**	Retinal periphlebitis/vasculitis and/or granulomatous choroidal involvement	CME, ERM, vitreomacular interface abnormalities, outer retinal changes, focal choroidal thickening, and hyporeflective elevated choroidal granulomas when present	Segmental or nodular periphlebitis, retinal vascular leakage, optic disc leakage, macular leakage, peripheral nonperfusion, and peripheral neovascularization, especially on ultrawidefield FA	Hypocyanescent choroidal lesions or dark dots corresponding to granulomatous choroidal involvement; may reveal subclinical choroidal disease	Focal or multifocal abnormalities reflecting RPE and outer retinal involvement; useful for mapping chronic chorioretinal changes	May show retinal or choriocapillaris flow abnormalities, but cannot assess leakage and is limited by segmentation error, signal attenuation, media opacity, and inter-device variability	Combination of retinal periphlebitis/vasculitis with choroidal granulomas or multifocal choroiditis supports sarcoid uveitis in the appropriate systemic context	Tuberculoma, tuberculosis-associated multifocal choroiditis, intraocular lymphoma, choroidal metastasis, idiopathic multifocal choroiditis, and other granulomatous choroidal lesions	**Active:** vascular leakage, optic disc leakage, CME, enlarging or active choroidal granulomas, ICGA hypocyanescent lesions. **Inactive/damage:** stable scars, RPE atrophy, chronic ERM, outer retinal loss, resolved leakage, and inactive chorioretinal lesions
**Vogt–Koyanagi–Harada disease**	Primary bilateral stromal choroiditis with secondary RPE and outer retinal	Serous retinal detachment, subretinal fluid, bacillary layer detachmen	Multifocal pinpoint leakage, pooling corresponding to serous retinal detachment, optic disc	Multiple hypocyanescent dark dots, diffuse choroidal stromal involvement, fuzzy choroidal	Acute or recurrent disease may show changes related to serous detachment and RPE	May show choriocapillaris flow deficits and retinal microvascular abnormalities, but	Bilateral diffuse choroidal thickening, serous retinal detachments, ICGA dark dots,	Sympathetic ophthalmia, posterior scleritis, central serous chorioretinopathy, inflammatory choriocapillariti	**Active:** subretinal fluid, diffuse choroidal thickening, ICGA dark dots, optic disc leakage, progressive

	involvement	t in some cases, diffuse choroidal thickening, choroidal folds, and outer retinal/RPE disruption in chronic disease	leakage, and late staining in active disease	vessels, and signs of occult choroiditis; especially useful for detecting persistent subclinical activity	stress; chronic disease may show mottled FAF abnormalities corresponding to RPE damage and sunset-glow fundus sequelae	quantitative OCTA metrics are not sufficiently standardized to guide treatment alone	and optic disc leakage favor VKH, especially without prior ocular trauma or surgery	s, APMPPE-like disease, and other causes of bilateral exudative retinal detachment	RPE/outer retinal changes. **Inactive/damage:** resolution of fluid, reduced choroidal activity, persistent RPE disturbance, outer retinal loss, chorioretinal atrophy, and sunset-glow fundus
**Sympathetic ophthalmia**	Bilateral stromal choroiditis/panuveitis, typically after penetrating ocular trauma or intraocular surgery	Serous retinal detachment, choroidal thickening, choroidal folds, hyperreflective septa, bacillary layer detachment in some cases, loss of normal choroidal architecture, and Dalen-Fuchs–type nodular changes	Optic disc leakage, multifocal posterior leakage, vascular leakage when retinal vasculitis is present, and staining or leakage related to active posterior inflammation	Hypocyanescent choroidal lesions or dark dots, choroidal stromal inflammation, and choroidal abnormalities overlapping with VKH	RPE disturbance in active or chronic disease; may highlight nummular atrophy, chronic chorioretinal scars, or peripapillary/retinal pigment epithelial sequelae	Adjunctive only; may document retinal or choriocapillaris flow abnormalities but does not reliably distinguish sympathetic ophthalmia from VKH and is limited in active inflammation	Bilateral VKH-like choroiditis in a patient with previous penetrating trauma or intraocular surgery strongly favors sympathetic ophthalmia	VKH, sarcoidosis, tuberculosis-associated choroiditis, syphilitic panuveitis, posterior scleritis, and other bilateral granulomatous panuveitides	**Active:** serous retinal detachment, choroidal thickening, optic disc leakage, ICGA choroidal lesions, active panuveitis. **Inactive/damage:** nummular chorioretinal atrophy, peripapillary subretinal fibrosis, widespread peripheral chorioretinal atrophy, RPE/outer retinal loss
**Tuberculosis-associated uveitis**	Phenotype-dependent:	Outer retinal and RPE	Retinal vascular leakage,	Choroidal or choriocapillaris	Active lesion borders may show	May demonstrate choriocapillar	Serpiginous-like choroiditis,	Serpiginous choroiditis, ampiginous	**Active:** expanding lesion borders,

	choroiditis, tuberculoma, serpiginous-like choroiditis, retinal vasculitis, panuveitis, or mixed retinochoroidal inflammation	disruption, choroidal thickening or granulomatous choroidal lesion in tuberculoma, structural changes at active lesion borders, subretinal fluid in selected cases, and chronic chorioretinal scarring	occlusive vasculitis, peripheral nonperfusion, optic disc leakage, inflammatory lesion activity, and neovascularization; ultrawidefield FA is useful for peripheral disease	abnormalities, often more extensive than clinically visible lesions; useful in choroiditis, tuberculoma, and serpiginous-like phenotypes	hyperautofluorescence or mixed autofluorescence; inactive scars often become hypoautofluorescent with RPE/chorioretinal atrophy	is or retinal flow abnormalities, but cannot establish tubercular etiology and cannot replace FA, ICGA, systemic testing, or microbiological correlation	tuberculoma, multifocal choroiditis, or retinal vasculitis in a compatible systemic/immunologic context favors tuberculosis-associated uveitis	choroiditis, sarcoid choroidal granuloma, syphilitic chorioretinitis, fungal chorioretinitis, lymphoma, metastasis, and noninfectious retinal vasculitis	active FAF changes, outer retinal/RPE disruption at lesion margins, vascular leakage, nonperfusion, neovascularization, vitritis, or paradoxical worsening. **Inactive/damage:** sharply demarcated scars, hypoautofluorescent atrophy, reduced leakage, stable chorioretinal scarring, RPE/outer retinal loss
**Syphilitic uveitis**	Predominantly retina, RPE, outer retina, and retinal vasculature; ASPPC is the most recognizable posterior phenotype	In ASPPC, outer retinal and ellipsoid zone disruption, granular or irregular RPE changes, possible subretinal material, and restoration of outer retinal architecture after	Late hyperfluorescence of placoid lesions, retinal vascular leakage, optic disc leakage, and vasculitis when present; ultrawidefield FA may reveal peripheral vascular involvement	Late hypofluorescence of placoid lesions in ASPPC; may help characterize choroidal or choriocapillaris involvement but is not diagnostic alone	Hyperautofluorescence corresponding to placoid posterior pole lesions; residual mottled FAF abnormalities may persist after structural damage	Adjunctive only; may show flow abnormalities but should not delay serologic confirmation or antimicrobial treatment; limited by artifacts and lack of validated disease-specific thresholds	Placoid yellow posterior pole lesion with hyper-FAF, outer retinal/EZ disruption on OCT, late FA hyperfluorescence, and late ICGA hypofluorescence strongly suggests ASPPC, but serologic confirmatio	APMPPE, MEWDS/AZOOR spectrum, viral retinitis, inflammatory choriocapillaritis, tuberculosis-associated choroiditis, VKH-like disease, lymphoma, and other masquerade inflammatory phenotypes	**Active:** placoid lesion, hyper-FAF, outer retinal/EZ disruption, FA leakage or late hyperfluorescence, ICGA hypofluorescence, retinal vasculitis, retinitis, or necrotizing retinitis. **Inactive/damage:** restoration of outer retinal layers when reversible; persistent RPE

	treatment when reversible; may also show findings of retinitis or necrotizing retinitis			n is mandatory	disturbance, outer retinal loss, chorioretinal atrophy, or residual FAF abnormalities

Abbreviations: APMPPE, acute posterior multifocal placoid pigment epitheliopathy; ASPPC, acute syphilitic posterior placoid chorioretinitis; AZOOR, acute zonal occult outer retinopathy; CME, cystoid macular edema; EDI-OCT, enhanced-depth imaging optical coherence tomography; ERM, epiretinal membrane; EZ, ellipsoid zone; FA, fluorescein angiography; FAF, fundus autofluorescence; ICGA, indocyanine green angiography; MEWDS, multiple evanescent white dot syndrome; OCT, optical coherence tomography; OCTA, optical coherence tomography angiography; RPE, retinal pigment epithelium; SS-OCT, swept-source optical coherence tomography; VKH, Vogt–Koyanagi–Harada disease.
